# Finite Buffer *GI*/*M*(*n*)/1 Queue with Bernoulli-Schedule Vacation Interruption under *N*-Policy

**DOI:** 10.1155/2014/392317

**Published:** 2014-10-29

**Authors:** P. Vijaya Laxmi, V. Suchitra

**Affiliations:** Department of Applied Mathematics, Andhra University, Visakhapatnam 530003, India

## Abstract

We study a finite buffer *N*-policy *GI/M(n)/*1 queue with Bernoulli-schedule vacation interruption. The server works with a slower rate during vacation period. At a service completion epoch during working vacation, if there are at least *N* customers present in the queue, the server interrupts vacation and otherwise continues the vacation. Using the supplementary variable technique and recursive method, we obtain the steady state system length distributions at prearrival and arbitrary epochs. Some special cases of the model, various performance measures, and cost analysis are discussed. Finally, parameter effect on the performance measures of the model is presented through numerical computations.

## 1. Introduction

In many real world queueing systems, the server may be unavailable for a random period of time when there is no customer in the waiting line at a service completion instant. This random period of server absence is often called server vacation; see Doshi [1] and Tian and Zhang [2]. The classical vacation scheme with Bernoulli-schedule (BS) discipline was introduced and studied by Keilson and Servi [3]. Various aspects of Bernoulli-schedule vacation models for single server queueing systems have been studied by Servi [4] and Ramaswamy and Servi [5].

Servi and Finn [6] introduced a class of semivacation policies known as working vacation (WV) wherein a customer is served at a slower rate rather than keeping completely inactive during a vacation. At a service completion epoch during a regular busy period if the queue length is empty, the server may take multiple working vacations (MWV). The analysis of *GI*/*M*/1 queue with MWV is carried out by Baba [7] using the matrix-analytic method. Analysis of finite buffer *GI*/*M*/1 queue with MWV can be found in Banik et al. [8]. In order to utilize the server effectively, vacation interruption (VI) has become an important aspect, where the server interrupts the vacation and resumes regular service if at least one customer is present in the queue at a service completion epoch during a vacation. Li and Tian [9] studied a Markovian queue with vacation interruption. The *GI*/*M*/1 queue with Bernoulli-schedule vacation interruption (BS-VI) has been analyzed by Tao et al. [10]. Using the matrix-analytic method, they have obtained the steady state distributions for the queue length, waiting time, and sojourn times.

For some controllable queueing systems with vacations, it is usually assumed that the server is available or unavailable completely depending upon the number of customers present in the system. Whenever the system is empty, the server goes on vacation. In the instant at which the server returns back from a vacation and finds at least *N* customers in the system, it begins serving immediately and exhaustively. This type of control policy is also called *N*-policy for the queueing systems with vacations. A brief analysis on finite buffer *GI*/*M*/1 queue with *N*-policy has been given by Ke and Wang [11] and Ke [12]. Tadj et al. [13] investigated a quorum queueing system with a random setup time under *N*-policy and with BS vacations. An infinite buffer Markovian queue with MWV and *N*-policy is generalized by Zhang and Xu [14].

State dependent queues are dependent on the queue size, arrivals, and their service times. This is applicable in many areas like cellular manufacturing cells, routers, and switches that regulate the transmission of information packages having finite buffer capacity. In many of these applications, the arrival and service rates depend on the state of the queue. These queues have applications on arrivals and service rates which yield less waiting time in the system. Chao and Rahman [15] have analyzed an *N*-policy *G*/*M*(*n*)/1/*K* queue with state dependent vacations. An efficient algorithm for the state dependent services and state dependent MWV for *GI*/*M*(*n*)/1/*N* has been presented by Goswami et al. [16]. Recently, a computational algorithm for the steady state probabilities in a *GI*/*M*(*n*)/1/*N*/SWV-VI queue has been presented by Laxmi et al. [17].

The present paper is an extension of the work of [10, 16] wherein our aim is to include *N*-policy and BS-VI in a finite buffer *GI*/*M*(*n*)/1 queue. The analysis of such state dependent service models has not been carried out so far to the best of our knowledge. Further, the inclusion of *N*-policy with BS-VI utilizes the server more and decreases the waiting lines effectively in order to economize operating cost and energy consumption.

Motivated by the above observations, this paper aims to contribute to the theory of BS-VI models with *N*-policy. The service times during service period, vacation period, and vacation times are exponentially distributed. We provide a recursive method using the supplementary variable technique and treating the remaining interarrival time as the supplementary variable, to develop the steady state system length distributions at prearrival and arbitrary epochs. The recursive method is powerful and easy to implement. Some performance measures such as blocking probability, the expected queue length, and the expected waiting time have been evaluated. Numerical results have been illustrated in the form of tables and graphs. A cost optimization problem is considered with a special case.

The paper is structured as follows. Next section presents the description and analysis of the model. Some special cases are derived in Section 3. Various performance measures and cost model are discussed in Section 4.  Section 5 contains some numerical results to show the effectiveness of the model parameters followed by conclusions in Section 6.

## 2. Model Description

In this paper, we consider a *GI*/*M*(*n*)/1/*K* queue with working vacations and BS-VI under *N*-policy, where *K* is the finite buffer space. We assume that the interarrival times of successive arrivals are independent and identically distributed (i.i.d.) random variables with cumulative distribution function *A*(*x*), probability density function *a*(*x*), *x* ≥ 0, and Laplace-Stieltjes transform (L.-S. T) *A* ^*^(*θ*) with mean interarrival time 1/*λ* = −*A* ^∗(1)^(0), where *h* ^(1)^(0) is the first derivative of *h*(*θ*) evaluated at *θ* = 0. The customers are served by a single server by first come first served rule. Whenever the system becomes empty, the server takes a WV. During a WV, a customer is served at a rate generally lower than the regular service rate. At a service completion epoch during WV, if there are at least *N* customers present in the queue, the server interrupts the vacation with probability $\bar{q}=1-q$ and switches to regular service and otherwise continues the vacation with probability *q*. The service times during regular busy period and during WV period are exponentially distributed with rates *μ*_*n*_ and *η*_*n*_, 1 ≤ *n* ≤ *K*, respectively, if there are *n* customers present in the system before the beginning of a service. The vacation times also follow exponential distribution with rate *γ*_*n*_, 1 ≤ *n* ≤ *K*, when there are *n* customers in the system. Let *μ*, *η*, and *γ* be the mean service rates during a regular busy period, during WV period and mean vacation rate respectively. They are given by *μ* = ∑_*n*=1_^*K*^ *μ*_*n*_/*K*, *η* = ∑_*n*=1_^*K*^ *η*_*n*_/*K*, and *γ* = ∑_*n*=1_^*K*^ *γ*_*n*_/*K*. The traffic intensity is given by *ρ* = *λ*/*μ*. Let us define the state of the system at time *t* as *N*_*s*_(*t*) denoting the number of customers present in the system including the one in service, *U*(*t*) is the remaining interarrival time for the next arrival, and

(1) $$\begin{array}{c} \zeta \left(t\right)=\left\{\begin{array}{cc} 0, & \text{if}\;\;\text{the}\;\;\text{server}\;\;\text{is}\;\;\text{in}\;\;\text{WV}\;\;\text{period},\\ 1, & \text{if}\;\;\text{the}\;\;\text{server}\;\;\text{is}\;\;\text{in}\;\;\text{regular}\;\;\text{busy}\;\;\text{period}. \end{array}\right. \end{array}$$

The joint probabilities denoted by *π*_*i*,0_(*x*, *t*) and *π*_*i*,1_(*x*, *t*) are defined as

(2) πn,jx,tdx  =lim⁡t→∞PNst≤n,x≤Ut≤x+dx,ζt=j,              x≥0, j≤n≤K, j=0,1.

The above probabilities at steady state are denoted by *π*_*n*,*j*_(*x*).

### 2.1. Analysis of the Model

The system length distributions at prearrival epoch are obtained by developing differential-difference equations at steady state. Treating the remaining interarrival time as supplementary variable, we write the equations as

(3) −π0,0(1)(x)=μ1π1,1(x)+η1π1,0(x),−πn,01x=−ηnπn,0x+axπn−1,00+ηn+1πn+1,0x, 1≤n≤N−1,−πn,01x=−αnπn,0x+axπn−1,00+qηn+1πn+1,0(x), N≤n≤K−1,−πK,01x=−αKπK,0x+axπK−1,00+πK,00,−π1,11x=−μ1π1,1x+μ2π2,1x,−πn,11x=−μnπn,1x+μn+1πn+1,1x+a(x)πn−1,1(0), 2≤n≤N−1,−πn,11x=−μnπn,1x+μn+1πn+1,1x+q¯ηn+1πn+1,0(x)+γnπn,0x+axπn−1,10, N≤n≤K−1,−πK,11x=−μKπK,1x+γKπK,0x+axπK−1,10+πK,10,

where *α*_*n*_ = *γ*_*n*_ + *η*_*n*_, *π*_*n*,*j*_(0), *j* = 0,1, are the respective rates of arrivals; that is, an arrival is about to occur. Let us define the Laplace transforms of *π*_*n*,*j*_(*x*) as *π*_*n*,j_^*^(*θ*) = ∫_0_^*∞*^ *e* ^−*θx*^ *π*_*n*,*j*_(*x*)*dx*, *Reθ* ≥ 0. Hence, *π*_*n*,*j*_ ≡ *π*_*n*,j_^*^(0) are the joint probabilities that there are *n* customers in the system and the server is in state *j*, *j* = 0,1. Multiplying the above set of equations by *e* ^−*θx*^ and integrating with respect to *x* from 0 to *∞* yield

(4) $$\begin{array}{c} -\theta \pi_{0,0}^{*}\left(\theta \right)=\mu_{1}\pi_{1,1}^{*}\left(\theta \right)+\eta_{1}\pi_{1,0}^{*}\left(\theta \right)-\pi_{0,0}\left(0\right), \end{array}$$

(5) ηn−θπn,0∗θ=ηn+1πn+1,0∗θ+A∗θπn−1,00−πn,00, 1≤n≤N−1,

(6) αn−θπn,0∗θ=qηn+1πn+1,0∗θ+A∗θπn−1,00−πn,00, N≤n≤K−1,

(7) αK−θπK,0∗θ=A∗θπK−1,00+πK,00−πK,00,

(8) $$\begin{array}{c} \left(\mu_{1}-\theta \right)\pi_{1,1}^{*}\left(\theta \right)=\mu_{2}\pi_{2,1}^{*}\left(\theta \right)-\pi_{1,1}\left(0\right), \end{array}$$

(9) μn−θπn,1∗θ=μn+1πn+1,1∗θ+A∗θπn−1,10−πn,10, 2≤n≤N−1,

(10) μn−θπn,1∗θ=μn+1πn+1,1∗θ+q¯ηn+1πn+1,0∗θ+γnπn,0∗θ+A∗θπn−1,10−πn,10, N≤n≤K−1,

(11) μK−θπK,1∗θ=γKπK,0∗θ+A∗θπK,10+πK−1,10−πK,10.

Further, adding (4) to (11) and taking limit as *θ* → 0, we obtain the following result:

(12) $$\begin{array}{c} \overset{K}{\underset{n=0}{\sum }}\pi_{n,0}\left(0\right)+\overset{K}{\underset{n=1}{\sum }}\pi_{n,1}\left(0\right)=\lambda . \end{array}$$

The left hand side denotes the mean number of entrances into the system per unit time and is equal to the mean arrival rate *λ*.

Solving the set of equations from (5) to (11) in a backward recursion we get the following expressions.

Substituting *θ* = *α*_*n*_ in (7) to (6) and *θ* = *η*_*n*_ in (5), we get

(13) πK−1,00=1−A∗αKA∗αKπK,00,πn−1,00=πn,00−qηn+1πn+1,0∗αnA∗αn, n=K−1,…,N,πn−1,0(0)=πn,00−ηn+1πn+1,0∗ηnA∗ηn, n=N−1,…,1,

where, for *θ* ≠ *α*_*n*_ and *θ* ≠ *η*_*n*_, the unknowns *π*_*n*,0_^*^(*θ*) are obtained from (7) to (5) as

(14) πK,0∗θ=A∗θ−A∗αKαK−θA∗αKπK,00,πn,0∗θ=qηn+1πn+1,0∗θ+A∗θπn−1,00−πn,00αn−θ,             n=K−1,…,N,πn,0∗θ=ηn+1πn+1,0∗θ+A∗θπn−1,00−πn,00ηn−θ,              n=N−1,…,2.

Substituting *θ* = *μ*_*n*_ in (11) to (9) we get

(15) πK−1,10=1−A∗μKA∗μKπK,10−γKA∗μKπK,0∗μK,πn−1,10=πn,10A∗μn−μn+1πn+1,1∗μnA∗μn−q¯ηn+1πn+1,1∗μnA∗μn−γnπn,0∗μnA∗μn, n=K−1,…,N,πn−1,1(0)=πn,10A∗μn−μn+1πn+1,1∗μnA∗μn, n=N−1,…,2.

For *θ* ≠ *μ*_*n*_, *π*_*n*,1_^*^(*θ*) are given by the following:

(16) πK,1∗θ =γKπK,0∗θ+A∗θπK−1,10+πK,10−πK,10μK−θ,πn,1∗θ=γnπn,0∗θ+q¯ηn+1πn+1,0∗θ+μn+1πn+1,1∗θμn−θ+A∗(θ)πn−1,1(0)−πn,1(0)(μn−θ), n=K−1,…,N,πn,1∗θ=μn+1πn+1,1∗θ+A∗θπn−1,10−πn,10μn−θ,             n=N−1,…,2,π1,1∗(θ)=μ2π2,1∗(θ)−π1,10μ1−θ.

Differentiating (5) to (7) and setting *θ* = *α*_*K*_ in (7), *θ* = *α*_*n*_ in (6), and *θ* = *η*_*n*_ in (5), we obtain, respectively,

(17) $$\begin{array}{c} \pi_{K,0}^{*}\left(\alpha_{K}\right)=-A^{*\left(1\right)}\left(\alpha_{K}\right)\left(\pi_{K-1,0}\left(0\right)+\pi_{K,0}\left(0\right)\right), \end{array}$$

(18) πn,0∗αn=−qηn+1πn+1,0∗1αn+A∗1αnπn−1,00,              n=K−1,…,N,

(19) $$\begin{array}{c} \pi_{n,0}^{*}\left(\eta_{n}\right)=-\left(\eta_{n+1}\pi_{n+1,0}^{*\left(1\right)}\left(\eta_{n}\right)+A^{*\left(1\right)}\left(\eta_{n}\right)\pi_{n-1,0}\left(0\right)\right). \end{array}$$

Similarly, differentiating (11) to (8) and setting *θ* = *μ*_*n*_  (1 ≤ *n* ≤ *K*), the expressions are given by

(20) πK,1∗μK=−γKπK,0∗1(μK)+A∗1μKπK−1,10+πK,10,πn,1∗μn=−γnπn,0∗1μn+q¯ηn+1πn+1,0∗1μn  + μn+1πn+1,0∗1μn+A∗1μnπn−1,10,                n=K−1,…,N,πn,1∗μn=−μn+1πn+1,0∗1μn+A∗1μnπn−1,10,             n=N−1,…,2,π1,1∗μ1=−μ2π2,1∗1μ1.

Using the above expressions one can evaluate *π*_*n*,*j*_(0), (*j* ≤ *n* ≤ *K*).

### 2.2. Relation between Steady State Distributions at Prearrival and Arbitrary Epochs

Let *π*_*n*,*j*_ ≡ *π*_*n*,j_^*^(0), *j* ≤ *n* ≤ *K*, *j* = 0,1, be the arbitrary and joint probabilities that there are *n* customers in the system and the server is in state *j*, *j* = 0,1, and let *π*_*n*,*j*_^−^ denote the prearrival epoch probabilities. Applying Bayes' theory and the result (12), we have

(21) $$\begin{array}{c} \pi_{n,j}^{-}=\frac{\pi_{n,j}\left(0\right)}{\lambda },\;j\leq n\leq K,\;\;j=0,1. \end{array}$$

To obtain the arbitrary epoch probabilities, we develop relations between prearrival and arbitrary epoch probabilities.

Setting *θ* = 0 in (7) to (5) and (11) to (9) and using (21), we obtain

(22) πK,0=λαKπK−1,0−,πn,0=λαnπn−1,0−+q¯ηn+1−γn+1αn+1πn,0−∑j=n+1K−1  +∑j=n+1K−1q¯ηj+1−γj+1αj+1×∏s=n+1jqηsαsπs,0−,           n=K−1, K−2,…,N,πn,0=ληnπn−1,0−+γNαNπN−1,0−+q¯ηN+1−γN+1αN+1πN,0−∑j=n+1K−1  +∑j=N+1K−1q¯ηj+1−γj+1αj+1∏s=N+1jqηsαsπs,0−,                n=N−1,…,1,πK,1=λμKγKαKπK−1,0−+πK−1,1−,πn,1=λμnπn−1,1−+γnαnπn−1,0−−∑j=nK−1q¯ηj+1−γj+1αj+1  ∑j=n+1K−1×∏s=njqs−nηsαsπs,0−, n=K−1, K−2,…,N,πn,1=λμnπn−1,1−+γNαNπN−1,0−−∑j=NK−1q¯ηj+1−γj+1αj+1  ∑j=n+1K−1×∏s=Njqs−iηsαsπs,0−,  n=N−1, N−2,…,2,π1,1=λμ1γNαNπN−1,0−−∑j=NK−1q¯ηj+1−γj+1αj+1  ∑j=n+1K−1×∏s=Njqs−iηsαsπs,0−.

Using the normalization condition, the only unknown *π*_0,0_ is obtained as

(23) $$\begin{array}{c} \pi_{0,0}=1-\overset{K}{\underset{n=1}{\sum }}\left(\pi_{n,0}+\pi_{n,1}\right). \end{array}$$

## 3. Special Cases

Some models available in the literature are deduced as special cases of our model by taking specific values of the parameters *N*, *q*, *η*_*n*_, *μ*_*n*_, and *γ*_*n*_.

**Case 1 .**

If *q* = *N* = 1, our model reduces to finite buffer *GI*/*M*(*n*)/1 queue with MWV. In this case (5) and (9) do not exist. Our results are found to match with the results available in Goswami et al. [16].

**Case 2 .**

If *η*_*n*_ → 0, the model becomes *GI*/*M*(*n*)/1/*K* with *N*-policy; our results are in accordance with Chao and Rahman [15].

**Case 3 .**

If ∀*n*, *μ*_*n*_ = *μ*, *γ*_*n*_ → *∞*, *η*_*n*_ → 0, *q* = 1, the model reduces to *GI*/*M*/1/*K* queue and the results match with the results available in Ke and Wang [11].

## 4. Performance Measures

In this section, some operating characteristics such as the average number of customers in the queue (*L*_*q*_) (system (*L*_*s*_)), the average waiting time of a customer in the queue (*W*_*q*_) (system (*W*_*s*_)), and the blocking probability of the server (*P*_loss_) are evaluated. They are, respectively, given by

(24) $$\begin{array}{c} L_{q}=\overset{K}{\underset{n=1}{\sum }}\left(n-1\right)\pi_{n,0}+\overset{K}{\underset{n=1}{\sum }}\left(n-1\right)\pi_{n,1},\\ L_{s}=\overset{K}{\underset{n=1}{\sum }}n\pi_{n,0}+\overset{K}{\underset{n=1}{\sum }}n\pi_{n,1},\\ P_{\text{loss}}=\pi_{K,0}^{-}+\pi_{K,1}^{-},\\ W_{q}=\frac{L_{q}}{\hat{\lambda }},\;\;W_{s}=\frac{L_{s}}{\hat{\lambda }}, \end{array}$$

where $\hat{\lambda }=\lambda (1-P_{\text{loss}})$ is the effective arrival rate.

### 4.1. Cost Model

In this subsection, we formulate an expected cost model, in which mean service rate during vacation *η* is the decision variable. Let us define the following:

 
*C*_*μ*_≡ cost per unit time during regular busy period,
 
*C*_*η*_≡ cost per unit time during working vacation period,
 
*C*_*lq*_≡ cost per unit time for a customer waiting in the queue,
 
*C*_ploss_≡ cost per unit time when a customer is lost due to blocking.

The total expected cost function per unit time is given by

(25) $$\begin{array}{c} \text{Minimize}:f\left(\eta \right)=C_{\mu }\mu +C_{\eta }\eta +C_{lq}L_{q}+C_{\text{ploss}}P_{\text{loss}}. \end{array}$$

Our objective is to determine the optimal mean service rate during vacation *η* ^*^ to minimize the cost function *f*(*η*). We employ the QFSM to solve the above optimization problem, as the computation of derivatives of the above expected cost function is a nontrivial task.

### 4.2. Quadratic Fit Search Method

Given a 3-point pattern, we can fit a quadratic function through corresponding functional values that has a unique minimum, *x* ^*q*^, for the given objective function *f*(*x*). Quadratic fit uses this approximation to improve the current 3-point pattern by replacing one of its points with approximate optimum *x* ^*q*^. The unique optimum *x* ^*q*^ of the quadratic function agreeing with *f*(*x*) at 3-point operation (*x* ^*l*^, *x* ^*m*^, *x* ^*h*^) occurs at

(26) xq≅12fxlxm2−xh2+fxmxm2−xh2+fxhxl−xm−1  ×xh2−xl2+fxhxl2−xm2  ×fxlxm−xh+fxmxh−xlxm2−xh2  xm2−xh2+fxhxl−xm−1.

## 5. Numerical Results

To validate the results obtained earlier, some numerical computations have been done and some of them are presented in the form of tables and graphs. The parameters of the system are taken as *K* = 10, *N* = 5, the traffic intensity *ρ* = 0.5, *μ*_*n*_ = ln⁡(*n* + 0.4), *η*_*n*_ = ln⁡(*n* + 0.2), and *γ*_*n*_ = ln⁡(*n* + 0.3) with mean values *μ* = 1.617224, *η* = 1.566202, and *γ* = 1.592235, respectively, unless otherwise mentioned separately in the respective graphs and tables. The various cost parameters are taken as *C*_*μ*_ = 20, *C*_*η*_ = 18, *C*_*lq*_ = 30, and *C*_ploss_ = 10.

Table 1 presents the performance characteristics of the *E*_2_/*M*(*n*)/1/10 model for different *q* values. It can be observed that as *q* increases, the system characteristics increase and model with VI (*q* = 0) performs better than the model without VI (*q* = 1) as expected in practice.

**Figure 1:**
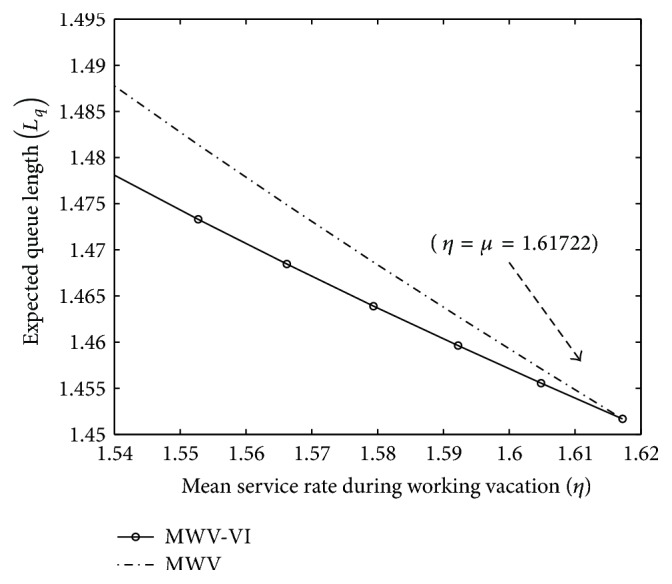
Impact of *η* on *L*_*q*_.

Figure 1 depicts the effect of *η* on the expected queue length (*L*_*q*_) in models with and without VI for *HE*_2_ interarrival time distribution with *λ*_1_ = 0.381248, *λ*_2_ = 3.2, *σ*_1_ = 0.4, and *σ*_2_ = 0.6. We observe that as *η* increases, *L*_*q*_ decreases. Further, in both models (MWV and MWV-VI) *L*_*q*_ converges to the same value as *η* approaches *μ*.

**Figure 2:**
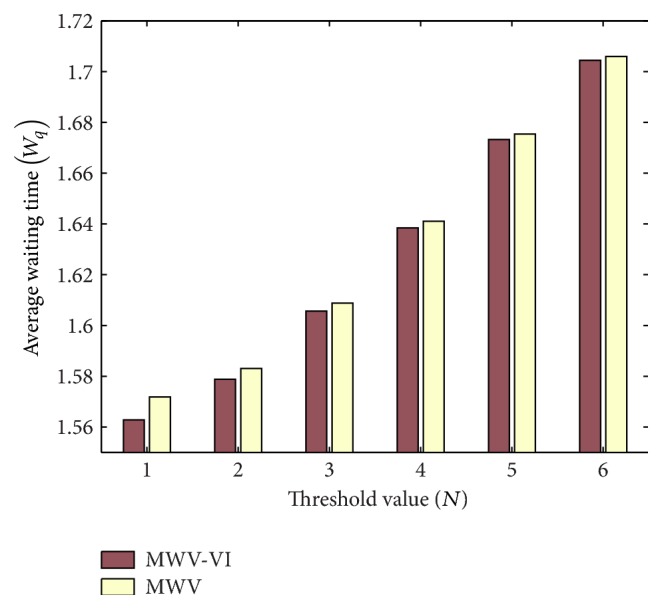
Effect on *N* on *W*_*q*_.

Figure 2 shows the impact of threshold value *N* on *W*_*q*_ when interarrival time is exponentially distributed. It is clear from the figure that *W*_*q*_ increases with the increase of *N*. This is because as *N* increases more customers are required for the service start-up that results in increase of waiting time. Further, the average waiting time in case of queues without VI is higher as compared to queues with VI particularly for smaller *N* values.

**Figure 3:**
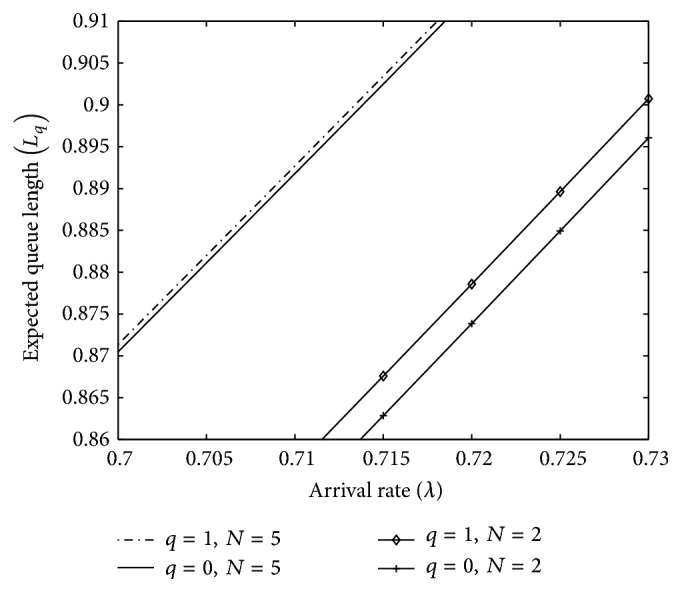
Impact of *λ* on *L*_*q*_.

A similar observation of above figure can be made from Figure 3 where the effect of *λ* on *L*_*q*_ for two different threshold values of *N* is shown. It is clear that as *N* increases, difference between two models for *L*_*q*_ reduces. Moreover we can observe that MWV-VI (*q* = 0) model gives us the better expected queue lengths.

**Figure 4:**
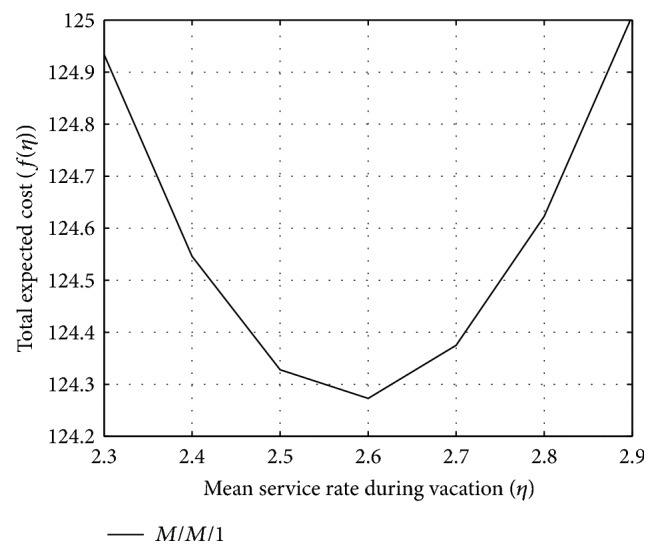
Effect on *η* on *f*(*η*).

The effect of *η* on the total expected cost function (*f*(*η*)) is shown in Figure 4, with constant service rates during regular busy period and during working vacation period and constant vacation rates. The state independent rates are chosen as *μ* = 2.8, *γ* = 0.9, and *λ* = 1.5. With the information of Figure 4, QFSM is applied by choosing the stopping tolerance *ϵ* = 10^−5^ and the initial 3-point pattern as (*η* ^*l*^, *η* ^*m*^, *η* ^*h*^) = (2.5,2.6,2.7). After six iterations, Table 2 shows that the minimum expected operating cost per unit time converges to the solution *f*(*η*) = 124.2712 for *η* ^*^ = 2.583980.

Table 3 describes the sensitivity analysis of cost function and the queue lengths between MWV and MWV-VI models for different *λ* and *N* values. The minimum expected cost and the average queue lengths increase with *N* and *λ* in both the models and the model with VI has lower queue lengths when the arrival rate is lower. The above observations highlight the fact that the model with MWV-VI has better performance than the MWV model for lower threshold value *N* and for *η* < *μ*.

## 6. Conclusions

In this paper, we have carried out an analysis of a renewal input state dependent *N*-policy queue with Bernoulli-schedule vacation interruption that has potential applications in production, manufacturing, traffic signals, telecommunication systems, and so forth. The Bernoulli-schedule parameter *q* enables combined study of the models with and without VI. Using the supplementary variable technique we have developed a recursive method to obtain the steady state system length distributions at various epochs. The recursive method used is powerful and easy to implement. Various performance measures are evaluated and a cost optimization problem is considered using quadratic fit search method. The method and analysis used in this paper can be applied to multiserver *GI*/*M*(*n*)/*C* and *MAP*/*M*(*n*)/*C* queues. These topics are left for future investigation.

## Figures and Tables

**Table 1:** Performance characteristics of *E*_2_/*M*(*n*)/1/10 queue with *N* = 5, λ = 0.808612.

	*q* = 0	*q* = 0.3	*q* = 0.6	*q* = 1
*L*_*q*_	1.222528	1.222661	1.222806	1.223020
*L*_*s*_	2.156477	2.156633	2.156803	2.157055
*P*_loss_	0.000027	0.000027	0.000027	0.000027
*W*_*q*_	1.511926	1.512090	1.512269	1.512534
*W*_*s*_	2.666959	2.667152	2.667362	2.667674

**Table 2:** Search for optimum service rate during working vacation period (η^*^).

η^*l*^	η^*m*^	η^*h*^	*f*(η^*l*^)	*f*(η^*m*^)	*f*(η^*h*^)	η^*q*^	*f*(η^*q*^)
2.50000	2.60000	2.70000	124.3275	124.2732	124.3748	2.58482	124.2712
2.50000	2.58482	2.60000	124.3275	124.2712	124.2732	2.58409	124.2712
2.50000	2.58409	2.58482	124.3275	124.2712	124.2712	2.58394	124.2712
2.50000	2.58394	2.58409	124.3275	124.2712	124.2712	2.58398	124.2712
2.58394	2.58398	2.58409	124.2712	124.2712	124.2712	2.58398	124.2712
2.58394	2.58398	2.58398	124.2712	124.2712	124.2712	—	—

**Table 3:** Sensitivity analysis of MWV and MWV-VI models.

*N*	λ		MWV			MWV-VI	
		η^*^	*f*(η)	*L*_*q*_	η^*^	*f*(η)	*L*_*q*_
1	1.1	0.138792	089.547341	1.034160	0.417693	086.290059	0.758794
	1.3	0.391248	099.693814	1.043741	0.560349	099.666960	1.122521

3	1.1	1.165482	101.761264	0.827047	0.947490	098.967713	0.823598
	1.3	1.455129	110.737740	0.951105	1.110389	107.311209	1.043741

5	1.1	1.843442	108.197096	0.633725	1.832134	107.507853	0.618745
	1.3	2.242303	116.015649	0.654932	2.147315	115.435968	0.692579
